# Supplementary material for: A molecular gradient along the longitudinal axis of the human hippocampus informs large-scale behavioral systems
Source: Nat Commun. 2020 Feb 19;11:960. doi: 10.1038/s41467-020-14518-3 (PMC7031290; doi:10.1038/s41467-020-14518-3)
Supplement: Supplementary file 1 — Supplementary Information [file 41467_2020_14518_MOESM1_ESM.pdf]

Supplementary Figures for A molecular gradient along the longitudinal axis of the human hippocampus informs large-scale behavioral systems

Jacob W. Vogel[a,*], Renaud La Joie[b], Michel J. Grothe[c], Alex Diaz-Papkovich[d], Andrew Doyle[a], Etienne Vachon-Presseau[e,f], Claude Lepage[a], Reinder Vos de Wael[a], Rhalena A. Thomas[a], Yasser Iturria-Medina[a], Boris Bernhardt[a], Gil D. Rabinovici[b], Alan C. Evans[a,*]

[a]*Montreal Neurological Institute, McGill University, Montréal, QC, Canada*
[b]*Memory and Aging Center, University of California, San Francisco, CA, USA*
[c]*German Center for Neurodegenerative Diseases (DZNE), Rostock, Germany*
[d]*McGill University and Genome Quebec Innovation Centre, Montréal, QC, Canada*
[e]*Faculty of Dentistry, McGill University*
[f]*Department of Anesthesia, Faculty of Medicine, McGill University*

1. Supplementary Figures

*Corresponding authors: jacob.vogel@mail.mcgill.ca, alan.evans@mcgill.ca

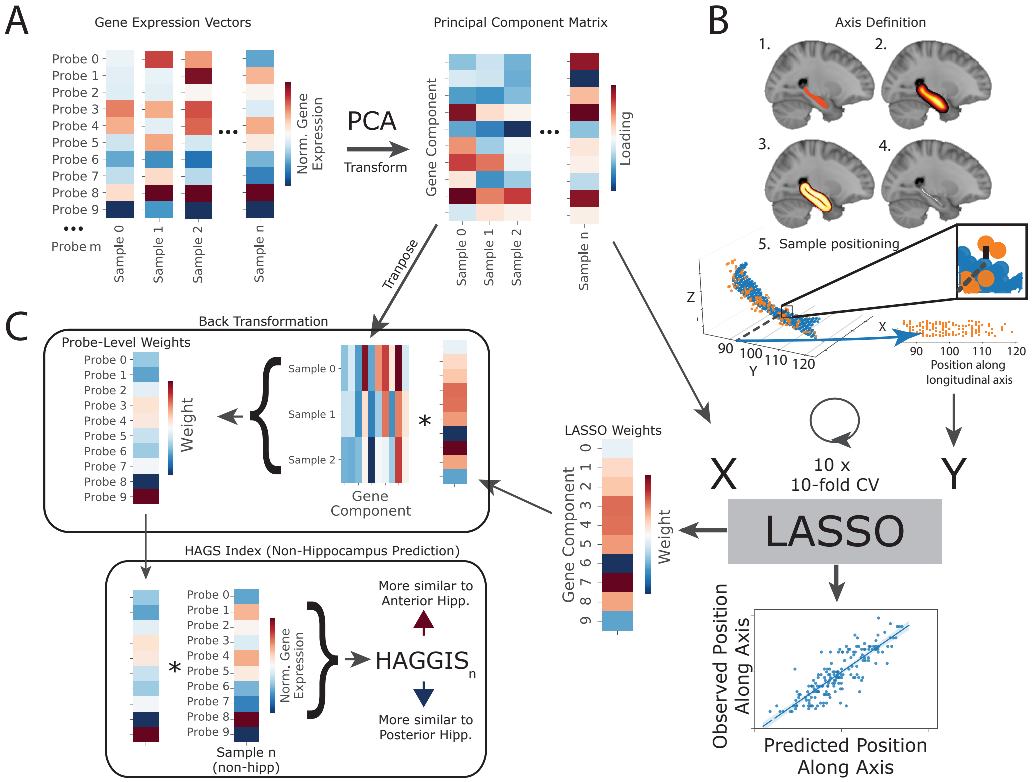

Supplementary Fig. 1: LASSO-PCR pipeline to predict the position of a tissue sample along the longitudinal axis of the hippocampus using gene expression. **(A)** The 170 (Sample) x 58,692 (probe) gene expression matrix was first reduced using principal components analysis (PCA), such that each sample had a singular value representing the loading onto each principal component. The principal component matrix was used as the predictor (X) variable in the LASSO-PCR model. **(B)** The longitudinal axis of the hippocampus was defined with a medial axis transform: 1) We start with a mask of the hippocampus, which is resampled to 0.5mm space. 2) The mask is dilated by creating a chamfer map measuring distance from the center of the hippocampus, extending out 10mm into a smooth hippocampus-shaped blob. 3) An inverse chamfer map was created inside the blob, local minimum of the derivatives of this map were computed. 4) These operations resulted in a hippocampus skeleton. 5) For each tissue sample (orange), the closest hippocampus skeleton voxel (blue) was located, and the y-axis of this coordinate was used as the position of the sample along the longitudinal axis, which was used as the dependent variable (Y). **(C)** A sparse LASSO regression model fit the (reduced) gene expression data to position along the atlas, with ten rounds of 10-fold cross-validation. Model weights were back-transformed to probe space. The back-transformed weights were applied to the gene expression vectors of non-hippocampus samples to the derive the HAGGIS, indicating genomic similarity to the anterior (positive) or posterior (negative) hippocampus.

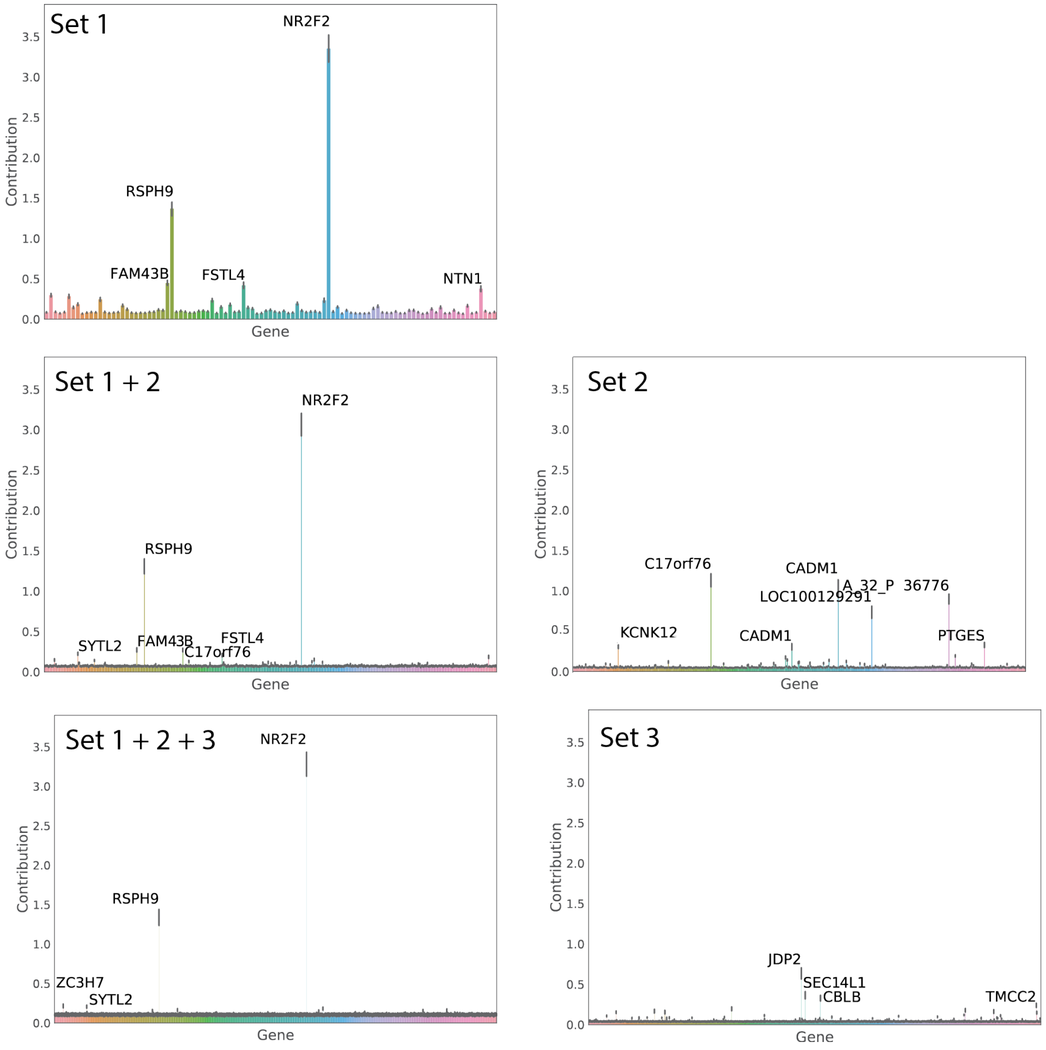

Supplementary Fig. 2: Feature-explainer applied to different gene sets. The Random-Forest based feature explainer was applied to different combinations of gene sets associated with position along the longitudinal axis of the hippocampus. For each plot, the y-axis represents local feature importance (and by extension, contribution to the model), indicating the degree to which, on average, perturbing the feature (probe) impacts individual model predictions. NR2F2 and RSPH9 consistently demonstrated the greatest importance when included in the model. Compared to Set 1, feature explainers identified more features with less importance for Sets 2 and 3. Error bars reperesent standard error of the mean

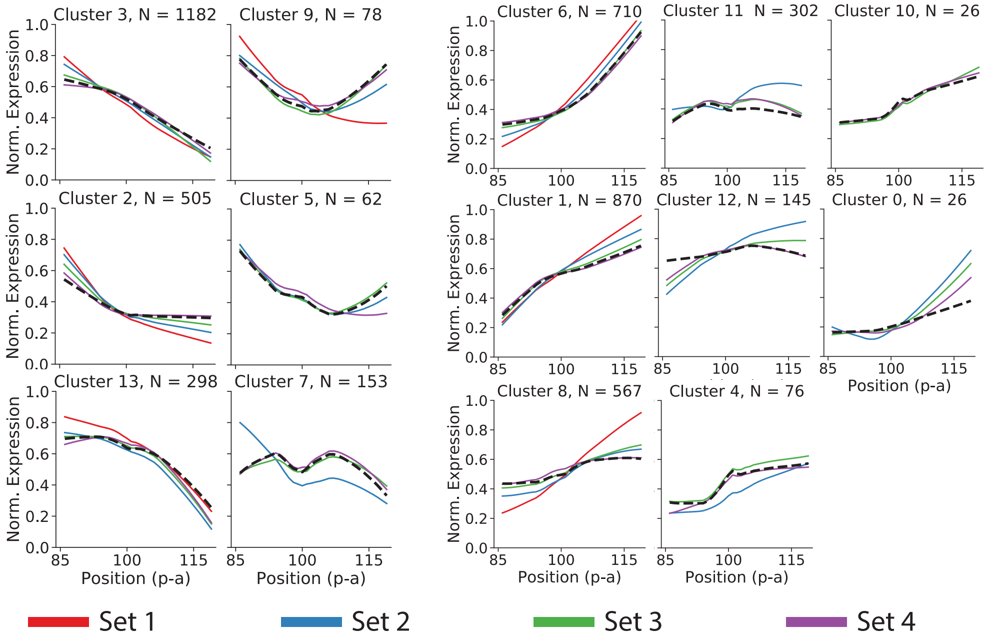

Supplementary Fig. 3: Expression pattern clusters. Fourteen clusters of normalized gene expression were identified across the 5000 genes in Sets 1, 2, 3 and 4. Each subpanel represents a cluster, and the subpanel heading includes the number of probes assigned to that cluster. For each cluster, the average normalized posterior-anterior expression pattern is visualized for the mean of all probes belonging to that cluster (black dashed), as well as the mean of all probes in each of Sets 1, 2, 3 and 4.

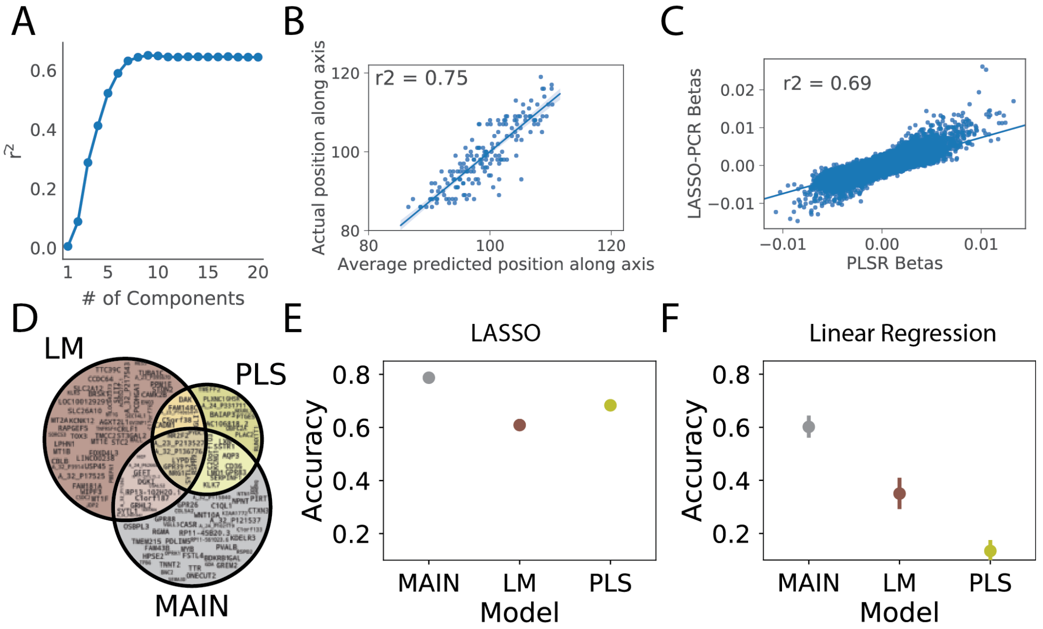

Supplementary Fig. 4: Validating results with PLSR. To ensure previous findings were not a product of algorithm choice, PLSR was fit to the gene expression data in order to predict position along the longitudinal axis of the hippocampus. **A** 10-fold cross-validation suggested nine as the optimal number of components. **B** Fitting the PLSR model to the data resulted in a similar r^2 as the LASSO-PCR approach. **C** The weights from the LASSO-PCR and PLSR models were highly correlated. **D** A Venn diagram showing overlap of the top 100 genes from the original LASSO-PCR model (MAIN), the PLSR model (PLS) and the top 100 genes ranked by correlation with longitudinal axis position (LM). textbfE 10-fold cross validation accuracy for a LASSO-based model similar to the model in the main text, when using the gene sets described in (D) as features. Confidence intervals were derived using bootstrapping. textbfF The same as (E) but using Linear Regression instead of a LASSO-based model. Error bars represent standard error of the mean cross-validation accuracy for runs across bootstrapped samples

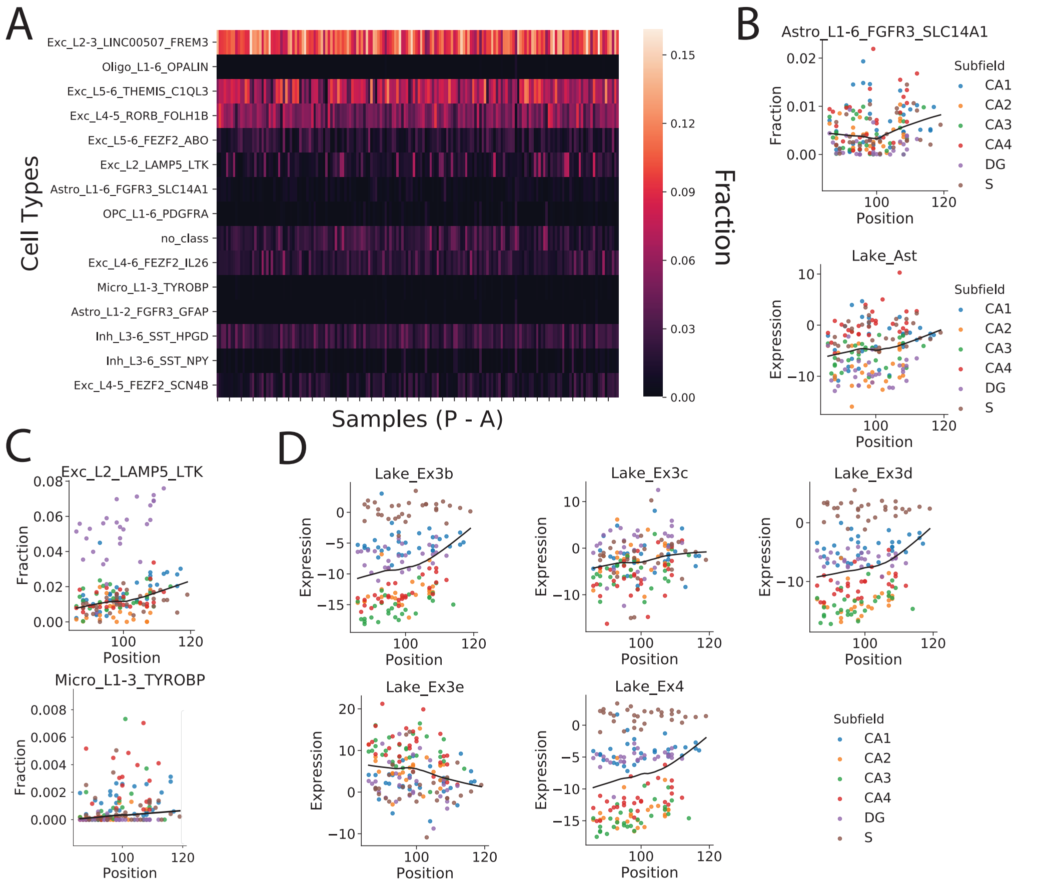

Supplementary Fig. 5: Analysis of cell type variation along the longitudinal axis of the hippocampus. **A** Estimated fractions for each of the 15 (reliably measured) cell types across all hippocampus samples, arrange from the most posterior to most anterior. **B** Estimated cell type fraction (above) and expression (below) of astrocytes across the two methods. Each dot is a sample (arranged posterior to anterior), and is colored by hippocampal subfield. A lowess curve is fitted over the data to show the approximate pattern of the cell type along the longitudinal axis. **C** Cell type proportion pattern for cell types associated with the longitudinal axis in the CiberSortX approach only. **D** Cell type proportion pattern for cell types associated with the longitudinal axis in the cell type expression approach only.

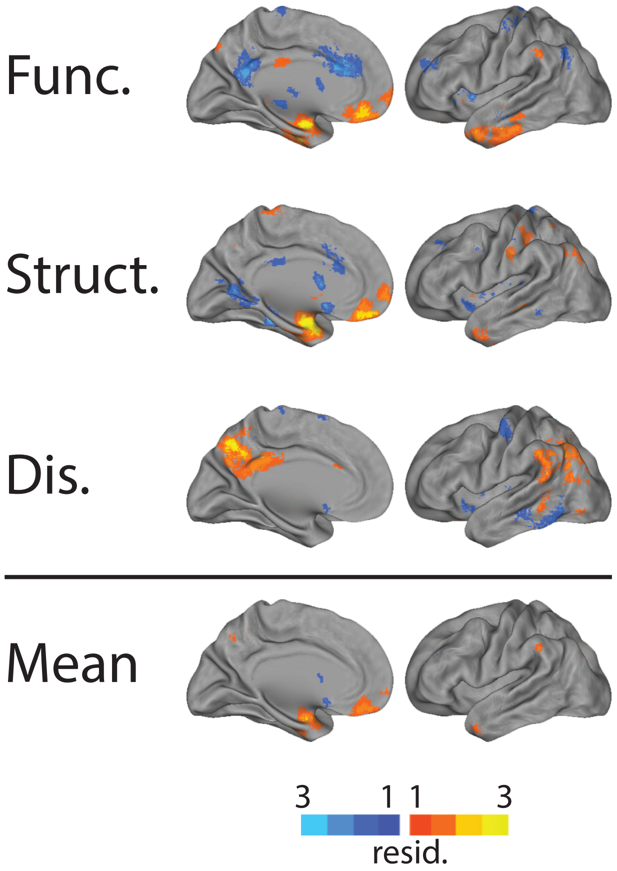

Supplementary Fig. 6: Disagreement between HAGGIS and cortico-hippocampal relationships. Each subpanel represents a cortical rendering of the residuals between HAGGIS and the cortico-hippocampal maps from the main text (Figure 5A), where the sign of the residual has been altered to reflect directionality. Specifically, orange colors represent disagreement in magnitude but not directionality, whereas blue colors represent disagreement in directionality. The average disagreement across all modalities is visualized at the bottom. The values are thresholded at 1 SD to highlight regions of most prominent disagreement.

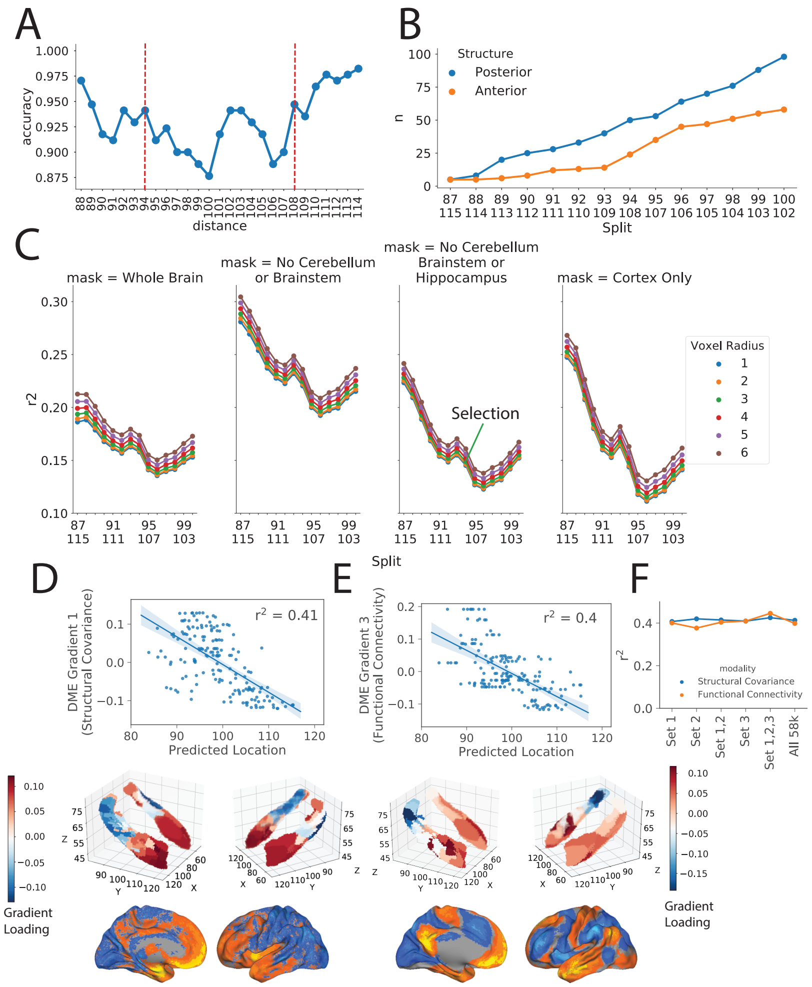

Supplementary Fig. 7: Validation of rsfMRI connectivity results. **(A)** An anterior-posterior split of the hippocampus was made at every y-coordinate along the hippocampal axis, and a Logistic Regression with HAGGIS was performed to classify anterior from posterior hippocampus. Accuracy at each split is visualized. The coordinates of the final split used for the analysis in the main text are indicated with red dashed lines. **(B)** The analysis was performed across several additional splits, indicated on the x-axis. The number of anterior and posterior samples included after each split are shown in orange and blue, respectively. The splits move from more extreme to more central as the x-axis moves from left to right. **C** The rsfmri analysis was repeated varying the radius of the extraction cube, the brain mask, and the anterior/posterior split. The r^2 of the correlation between HAGGIS and functional connectivity for each condition is shown. Diffusion map embedding was used to summarize principal axes of whole-brain functional connectivity (**D**) and structural covariance (**E**). Select gradients are correlated with the gene expression pattern predicting longitudinal axis location. The gradients are rendered onto a hippocampus surface, and expression of the gradient in whole-brain connectivity/covariance patterns is visualized. **F** The r^2 of relationships shown in C and D where the gene expression pattern is composed of different gene sets.

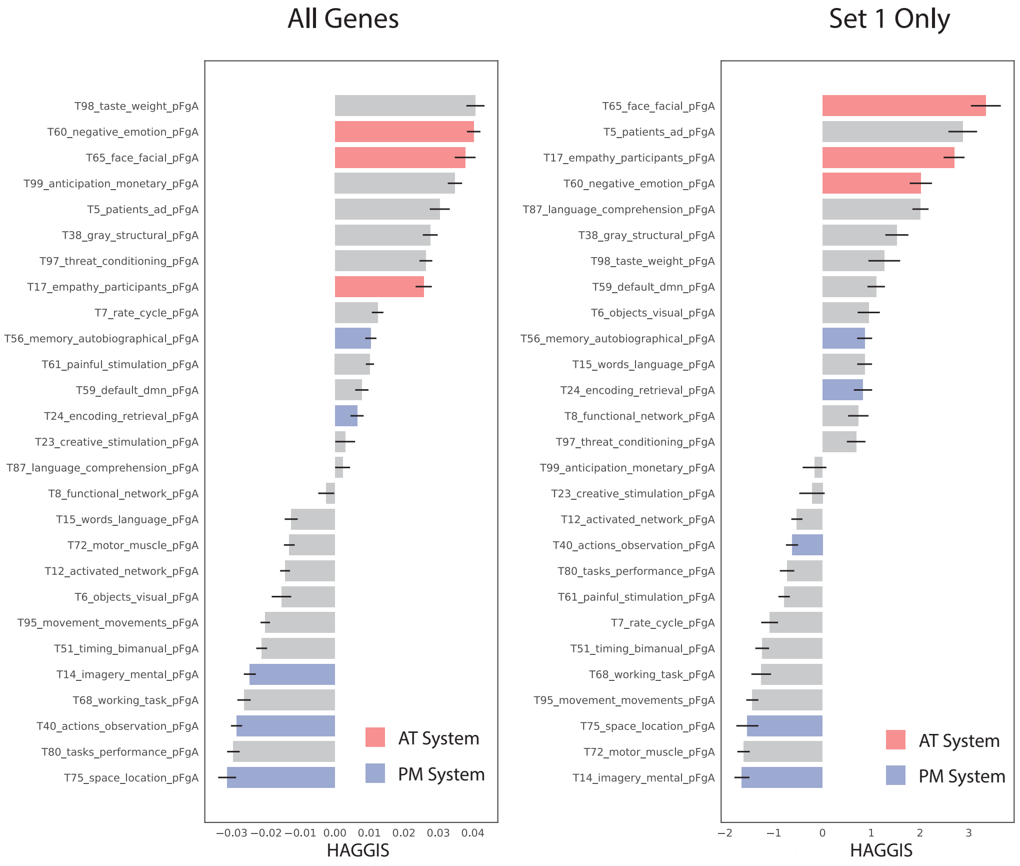

Supplementary Fig. 8: Cognitive meta-analysis when using all probes vs. top 100 probes. On the left is a vertical reproduction of main text Figure 7. On the right is the results of the exact same analysis, except calculating the HAGGIS using only the top 100 probes, rather than all 58,692 probes. The pattern is remarkably similar, especially as pertaining to the topics associated with the AT/PM system. Error bars represent standard deviation from the mean.

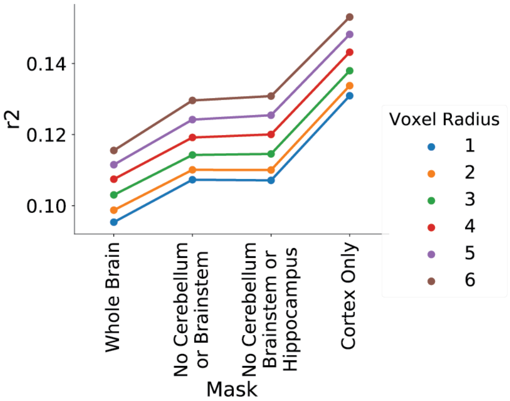

Supplementary Fig. 9: Validation of FDG neurodegeneration results. The analysis comparing HAGGIS to relative neurodegeneration in AD vs FTD was repeated using different extraction cube sizes and different brain masks. The r^2 for each condition is visualized

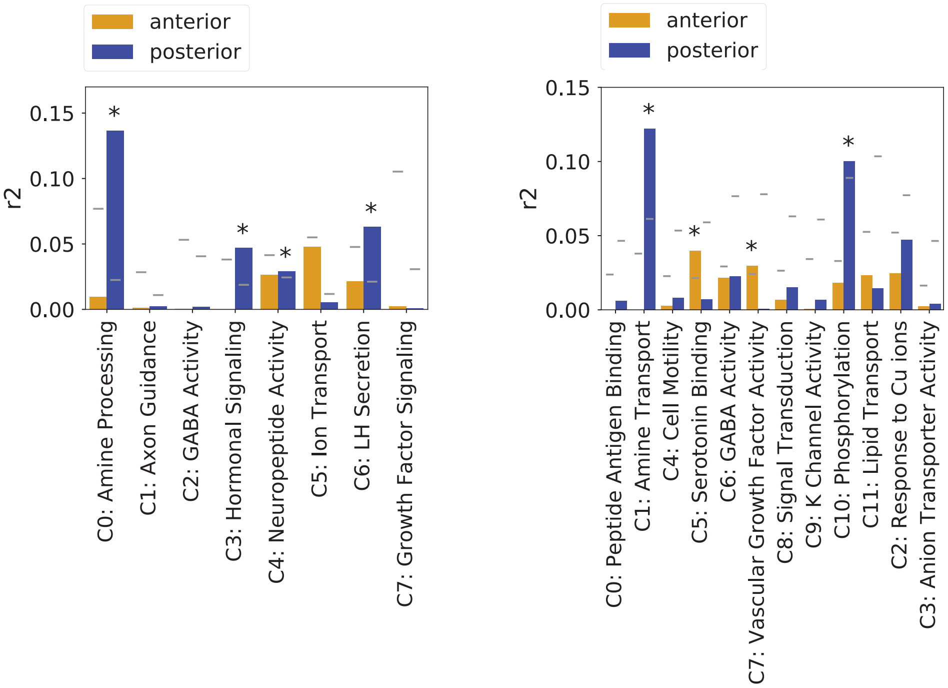

Supplementary Fig. 10: Association between gene ontology clusters and relative disease vulnerability. R^2 representing the relationship between each gene ontology cluster score from Set 2 (left) and Set 3 (right) and relative disease vulnerability. Gray lines indicate the 95% confidence interval of the null model for each cluster. Stars indicate greater then chance associations.

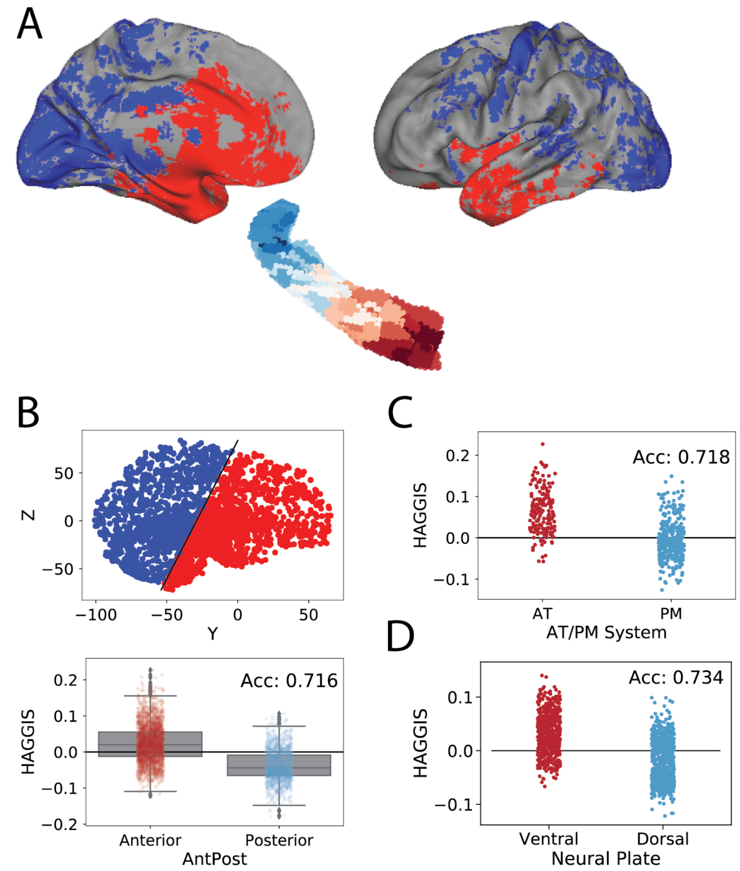

Supplementary Fig. 11: Agreement between HAGGIS pattern and other gradient-like neural phenomena. **(A)** Summary of cortical regions genomically related to anterior (red) and posterior (blue) hippocampus. **(B)** We differentiate brain samples as anterior or posterior by drawing a vertical line along the dorsal axis of the brainstem. Using a HAGGIS value of 0 as a classifier, we can discriminate 71.6% of samples accurately. **(C)** Samples were divided up into AT and PM regions based on [1]. A HAGGIS value of 0 differentiated samples with a 71.8% accuracy. **(C)** Sub-cortical samples were divided based on their presence within the ventral (basal medulla, pons, tegmentum, hypothalamus) or dorsal (alar medulla, cerebellum, tectum, thalamus) plate of the neural tube. A HAGGIS value of 0 differentiated samples with 73.4% accuracy. For boxplots, the center line, boxes and whiskers represent the median, inner quartiles, and rest of the data distribution (except outliers), respectively.

References

1. Ranganath, C. & Ritchey, M. Two cortical systems for memory-guided behaviour. *Nature Reviews Neuroscience* **13,** 713–726. ISSN: 1471003X. http://dx.doi.org/10.1038/nrn3338 (2012).
