# Supplementary material for: A molecular gradient along the longitudinal axis of the human hippocampus informs large-scale behavioral systems
Source: Nat Commun. 2020 Feb 19;11:960. doi: 10.1038/s41467-020-14518-3 (PMC7031290; doi:10.1038/s41467-020-14518-3)
Supplement: Supplementary file 3 — Reporting Summary [file 41467_2020_14518_MOESM3_ESM.pdf]

nature research

Corresponding author(s): Jacob W Vogel, Alan C. Evans

Last updated by author(s): October 14, 2019

Reporting Summary

Nature Research wishes to improve the reproducibility of the work that we publish. This form provides structure for consistency and transparency in reporting. For further information on Nature Research policies, see Authors & Referees and the Editorial Policy Checklist.

Statistics

For all statistical analyses, confirm that the following items are present in the figure legend, table legend, main text, or Methods section.

n/a	Confirmed	
☐	☒	The exact sample size (*n*) for each experimental group/condition, given as a discrete number and unit of measurement
☐	☒	A statement on whether measurements were taken from distinct samples or whether the same sample was measured repeatedly
☐	☒	The statistical test(s) used AND whether they are one- or two-sided<br>*Only common tests should be described solely by name; describe more complex techniques in the Methods section.*
☐	☒	A description of all covariates tested
☐	☒	A description of any assumptions or corrections, such as tests of normality and adjustment for multiple comparisons
☐	☒	A full description of the statistical parameters including central tendency (e.g. means) or other basic estimates (e.g. regression coefficient) AND variation (e.g. standard deviation) or associated estimates of uncertainty (e.g. confidence intervals)
☐	☒	For null hypothesis testing, the test statistic (e.g. *F*, *t*, *r*) with confidence intervals, effect sizes, degrees of freedom and *P* value noted<br>*Give P values as exact values whenever suitable.*
☒	☐	For Bayesian analysis, information on the choice of priors and Markov chain Monte Carlo settings
☒	☐	For hierarchical and complex designs, identification of the appropriate level for tests and full reporting of outcomes
☐	☒	Estimates of effect sizes (e.g. Cohen's *d*, Pearson's *r*), indicating how they were calculated

Our web collection on statistics for biologists contains articles on many of the points above.

Software and code

Policy information about availability of computer code

Data collection	No code was used to *collect* the data -- it was downloaded from websites or collected using traditional means. All code used to *prepare* data for analysis are explicitly documented and shared here: https://github.com/illdopejake/Hippocampus_AP_Axis. The enclosed code and scripts all used Python v. 3.6.2, except for one, which uses MINC Toolkit v. 1.0.08
Data analysis	All data analysis was completed using Python 3.6.2. The code used for all analyses in the manuscript are documented and shared here: https://github.com/illdopejake/Hippocampus_AP_Axis

For manuscripts utilizing custom algorithms or software that are central to the research but not yet described in published literature, software must be made available to editors/reviewers. We strongly encourage code deposition in a community repository (e.g. GitHub). See the Nature Research guidelines for submitting code & software for further information.

Data

Policy information about availability of data

All manuscripts must include a data availability statement. This statement should provide the following information, where applicable:

- Accession codes, unique identifiers, or web links for publicly available datasets
- A list of figures that have associated raw data
- A description of any restrictions on data availability

The datasets generated and/or analysed during the current study are available and summarized at https://github.com/illdopejake/Hippocampus_AP_Axis and Supplementary Table 8 of this manuscript, which includes all weblinks for further information, download instruction, license information and ethical compliance for each dataset.

Field-specific reporting

Please select the one below that is the best fit for your research. If you are not sure, read the appropriate sections before making your selection.

☒ Life sciences ☐ Behavioural & social sciences ☐ Ecological, evolutionary & environmental sciences

For a reference copy of the document with all sections, see nature.com/documents/nr-reporting-summary-flat.pdf

Life sciences study design

All studies must disclose on these points even when the disclosure is negative.

Sample size	Gene expression (Allen Human Brain Atlas): 3702 samples extracted from the brains of n=6 donors<br>Gene expression (Brainspan): n=4 donors<br>Functional connectivity (Brain GSP): n=1000<br>Structural covariance (OASIS): n = 153<br>FDG-PET: n = 70 (35 AD/ 35 FTD)<br>Functional coactivation meta-analysis: 11,406 articles
Data exclusions	Gene expression (Allen Human Brain Atlas): No data excluded<br>Brainspan: No data excluded<br>Functional connectivity (Brain GSP): No data excluded<br>Structural covariance (OASIS): All individuals who were not healthy controls, or who were 40 or older, we excluded.<br>FDG-PET: 5 patients with incomplete PET data excluded
Replication	The initial models were run using repeated 10-fold cross-validation. Leave-one-donor-out and leave-one-subfield-out models were also fit. Imaging results converge across several different modalities and datasets.
Randomization	All participants were used for all analyses. The only group comparisons compared patients with different types of neurodegenerative dementias (AD & FTD). Ten-fold cross-validation was used for some analyses. In such cases, participants were randomized to folds wth psuedorandom number generators, and this analysis was repeated several times, taking the mean results of all randomizations.
Blinding	Blinding is not relevant to this study, as analyses took place using several large public datasets, mostly of young healthy individuals. Group assignment did not take place, with the exception of AD and FTD patients. In no cases were outcomes dependent on participant grouping.

Reporting for specific materials, systems and methods

We require information from authors about some types of materials, experimental systems and methods used in many studies. Here, indicate whether each material, system or method listed is relevant to your study. If you are not sure if a list item applies to your research, read the appropriate section before selecting a response.

Materials & experimental systems

n/a	Involved in the study	
☒	☐	Antibodies
☒	☐	Eukaryotic cell lines
☒	☐	Palaeontology
☒	☐	Animals and other organisms
☐	☒	Human research participants
☒	☐	Clinical data

Methods

n/a	Involved in the study	
☒	☐	ChIP-seq
☒	☐	Flow cytometry
☐	☒	MRI-based neuroimaging

Human research participants

Policy information about studies involving human research participants

Population characteristics	FDG-PET scans were acquired from 35 patients with Alzheimer's disease (mean age = 62.0; 34% female; mean education = 16.1 years) and 35 patients with behavioral variant frontotemporal dementia or semantic variant primary progressive aphasia (mean age = 61.4; 54% female; mean education = 16.3 years). Characteristics for the OASIS, Brain GSP and Allen Human Brain Atlas datasets can be found at their respective websites (see above).
Recruitment	All public datasets were selected so as to represent normative (i.e. young/healthy) populations. The AD and FTD patients were recruited at a tertiary memory clinic at which they sought clinical care.
Ethics oversight	For AD and FDG patients, Informed consent was obtained from all subjects or their assigned surrogate decision-makers, and UCSF, University of California Berkeley, and the Lawrence Berkeley National Laboratory (LBNL) institutional review boards for

human research approved the study. Ethics approval for the OASIS, Brain GSP and Allen Human Brain Atlas datasets can be found at their respective websites (see above).

Note that full information on the approval of the study protocol must also be provided in the manuscript.

Magnetic resonance imaging

Experimental design

Design type	Resting state and task-based meta-analysis
Design specifications	n/a -- Meta-analyses assess thousands of studies with varied designs
Behavioral performance measures	n/a (see above)

Acquisition

Imaging type(s)	Functional, structural
Field strength	Brain GSP: 3T<br>OASIS: 1.5T
Sequence & imaging parameters	OASIS: T1-Weighted MPRage. Parameters can be found here (Table 1): https://www.mitpressjournals.org/doi/pdfplus/10.1162/jocn.2007.19.9.1498?casa_token=Yl6Ocw5kkl4AAAAA:HUZqmvTcgAjY4sb-poXOtIyVo8SLp3OnrJ6pWXC3Qn5I-ENzm0OpIdh19MV0Q2IpdLe4ZFdVado<br>Brain GSP: gradient-echo echo-planar imaging. All parameters from Dicom headers can be found here: https://www.nature.com/articles/sdata201531#s5 (Supplementary Table 5)
Area of acquisition	Whole-brain

Diffusion MRI ☐ Used ☒ Not used

Preprocessing

Preprocessing software	Public datasets used for the analyses were preprocessed derivatives. Detailed processing descriptions for each dataset can be found at it's respective website.<br><br>For FDG-PET data, six five-minute frames were realigned and averaged, and the average image was coregistered onto patient specic anatomical T1-MRI scans. Standard uptake value ratios (SUVR) were calculated using the pons (Freesurfer segmentation of the brainstem with manual cleaning) as a reference region.
Normalization	For FDG-PET, SUVR images were warped to the MNI template using MRI-derived parameters.<br><br>Allen Brain Atlas Data sample coordinates were also normalized using procedures described here: http://doi.org/10.5281/zenodo.2483290.
Normalization template	MNI ICBM152
Noise and artifact removal	Detailed processing descriptions for each dataset can be found at it's respective website (see above)
Volume censoring	see above

Statistical modeling & inference

Model type and settings	* A LASSO Principal Component Regression model is applied to learn gene expression features associated with sample location.<br>* Correlations are used to find associations between gene expression properties and functional connectivity and structural covariance with the hippocampus, as well as disease-specific neurodegeneration measured with FDG-PET<br>* Diffusion map embedding is used to summarize patterns of brain-hippocampus functional connectivity and structural covariance.
Effect(s) tested	See above

Specify type of analysis: ☒ Whole brain ☐ ROI-based ☐ Both

Statistic type for inference (See Eklund et al. 2016)	n/a
Correction	n/a, but cross-validation is performed.

Models & analysis

n/a	Involved in the study
☐	☒ Functional and/or effective connectivity
☐	☐ Graph analysis
☐	☒ Multivariate modeling or predictive analysis

Functional and/or effective connectivity	Functional connectivity measured as correlation of timeseries
Graph analysis	*Report the dependent variable and connectivity measure, specifying weighted graph or binarized graph, subject- or group-level, and the global and/or node summaries used (e.g. clustering coefficient, efficiency, etc.).*
Multivariate modeling and predictive analysis	LASSO-PCR is conducted where independent variables are gene expression patterns across ~58k genes. Principal components are used to reduce the dimensions and enter it into the LASSO model. Repeat 10-fold cross-validation is used, and the metric of evaluation is r-square.
