# Supplementary material for: A molecular gradient along the longitudinal axis of the human hippocampus informs large-scale behavioral systems
Source: Nat Commun. 2020 Feb 19;11:960. doi: 10.1038/s41467-020-14518-3 (PMC7031290; doi:10.1038/s41467-020-14518-3)
Supplement: Supplementary file 4 — Description of Additional Supplementary Files [file 41467_2020_14518_MOESM4_ESM.docx]

**Description of Additional Supplementary Files**

**Supplementary Data 1:** Feature weights for the top 100 features from the main (LASSO-PCR) model, PLSR model, and correlation ranked model.

**Supplementary Data 2:** Anterior-posterior expression cluster membership for each of the probes in Sets 1, 2, 3 and 4.

**Supplementary Data 3**: Gene ontology analysis clusters for all probes in Set 2

**Supplementary Data 4:** Gene ontology analysis clusters for all probes in Set 3.

**Supplementary Data 5:** Results from statistical comparisons between cell type expression, long axis position, and hippocampal subfields, for both cell type approaches.

**Supplementary Data 6:** Associations between all diffusion map embedding gradients and observed and predicted long axis position, for both functional and structural data.

**Supplementary Data 7:** Demographic data for brain tissue donors from both the Allen Human Brain Atlas and the BrainSpan dataset.

**Supplementary Data 8:** Summary of all datasets used in the manuscript, and their associated access links.

**Supplementary Data 9:** Index pointing to the Jupyter notebook where the analyses described in each subsection of the Methods can be found.
